# The echo chamber effect on social media

**DOI:** 10.1073/pnas.2023301118

**Published:** 2021-02-23

**Authors:** Matteo Cinelli, Gianmarco De Francisci Morales, Alessandro Galeazzi, Walter Quattrociocchi, Michele Starnini

**Affiliations:** ^a^Department of Environmental Sciences, Informatics and Statistics, Ca’Foscari Univerity of Venice, 30172 Venice, Italy;; ^b^Institute for Scientific Interchange (ISI) Foundation, 10126 Torino, Italy;; ^c^Department of Information Engineering, University of Brescia, 25123 Brescia, Italy;; ^d^Department of Computer Science, Sapienza University of Rome, 00185 Rome, Italy

**Keywords:** information spreading, echo chambers, social media, polarization

## Abstract

Social media may limit the exposure to diverse perspectives and favor the formation of groups of like-minded users framing and reinforcing a shared narrative, that is, echo chambers. However, the interaction paradigms among users and feed algorithms greatly vary across social media platforms. This paper explores the key differences between the main social media platforms and how they are likely to influence information spreading and echo chambers’ formation. We perform a comparative analysis of more than 100 million pieces of content concerning several controversial topics (e.g., gun control, vaccination, abortion) from Gab, Facebook, Reddit, and Twitter. We quantify echo chambers over social media by two main ingredients: 1) homophily in the interaction networks and 2) bias in the information diffusion toward like-minded peers. Our results show that the aggregation of users in homophilic clusters dominate online interactions on Facebook and Twitter. We conclude the paper by directly comparing news consumption on Facebook and Reddit, finding higher segregation on Facebook.

## Significance

We explore the key differences between the main social media platforms and how they are likely to influence information spreading and the formation of echo chambers. To assess the different dynamics, we perform a comparative analysis on more than 100 million pieces of content concerning controversial topics (e.g., gun control, vaccination, abortion) from Gab, Facebook, Reddit, and Twitter. The analysis focuses on two main dimensions: 1) homophily in the interaction networks and 2) bias in the information diffusion toward like-minded peers. Our results show that the aggregation in homophilic clusters of users dominates online dynamics. However, a direct comparison of news consumption on Facebook and Reddit shows higher segregation on Facebook.

Social media radically changed the mechanism by which we access information and form our opinions (1–5). We need to understand how people seek or avoid information and how those decisions affect their behavior (6), especially when the news cycle—dominated by the disintermediated diffusion of information—alters the way information is consumed and reported on. A recent study (7) limited to Twitter claimed that fake news travels faster than real news. However, a multitude of factors affects information spreading on social media platforms. Online polarization, for instance, may foster misinformation spreading (1, 8). Our attention span remains limited (9, 10), and feed algorithms might limit our selection process by suggesting contents similar to the ones we are usually exposed to (11–13). Furthermore, users show a tendency to favor information adhering to their beliefs and join groups formed around a shared narrative, that is, echo chambers (1, 14–18). We can broadly define echo chambers as environments in which the opinion, political leaning, or belief of users about a topic gets reinforced due to repeated interactions with peers or sources having similar tendencies and attitudes. Selective exposure (19) and confirmation bias (20) (i.e., the tendency to seek information adhering to preexisting opinions) may explain the emergence of echo chambers on social media (1, 17, 21, 22).

According to group polarization theory (23), an echo chamber can act as a mechanism to reinforce an existing opinion within a group and, as a result, move the entire group toward more extreme positions. Echo chambers have been shown to exist in various forms of online media such as blogs (24), forums (25), and social media sites (26–28). Some studies point out echo chambers as an emerging effect of human tendencies, such as selective exposure, contagion, and group polarization (13, 23, 29–31). However, recently, the effects and the very existence of echo chambers have been questioned (2, 27, 32). This issue is also fueled by the scarcity of comparative studies on social media, especially concerning news consumption (33). In this context, the debate around echo chambers is fundamental to understanding social media’s influence on information consumption and public opinion formation. In this paper, we explore the key differences between social media platforms and how they are likely to influence the formation of echo chambers or not. As recently shown in the case of selective exposure to news outlets, studies considering multiple platforms can offer a fresh view on long-debated problems (34). Different platforms offer different interaction paradigms to users, ranging from retweets and mentions on Twitter to likes and comments in groups on Facebook, thus triggering very different social dynamics (35). We introduce an operational definition of echo chambers to provide a common methodological ground to explore how different platforms influence their formation. In particular, we operationalize the two common elements that characterize echo chambers into observables that can be quantified and empirically measured, namely, 1) the inference of the user’s leaning for a specific topic (e.g., politics, vaccines) and 2) the structure of their social interactions on the platform. Then, we use these elements to assess echo chambers’ presence by looking at two different aspects: 1) homophily in interactions concerning a specific topic and 2) bias in information diffusion from like-minded sources. We focus our analysis on multiple platforms: Facebook, Twitter, Reddit, and Gab. These platforms present similar features and functionalities (e.g., they all allow social feedback actions such as likes or upvotes) and design (e.g., Gab is similar to Twitter) but also distinctive features (e.g., Reddit is structured in communities of interest called subreddits). Reddit is one of the most visited websites worldwide (https://www.alexa.com/siteinfo/reddit.com) and is organized as a forum to collect discussions on a wide range of topics, from politics to emotional support. Gab claims to be a social platform aimed at protecting freedom of speech. However, low moderation and regulation on content has resulted in widespread hate speech. For these reasons, it has been repeatedly suspended by its service provider, and its mobile app has been banned from both App and Play stores (36). Overall, we account for the interactions of more than 1 million active users on the four platforms, for a total of more than 100 million unique pieces of content, including posts and social interactions. Our analysis shows that platforms organized around social networks and news feed algorithms, such as Facebook and Twitter, favor the emergence of echo chambers.

We conclude the paper by directly comparing news consumption on Facebook and Reddit, finding higher segregation on Facebook than on Reddit.

## Characterizing Echo Chambers in Social Media

### Operational Definitions.

To explore the key differences between social media platforms and how they influence echo chambers’ formation, we need to operationalize a definition for them. First, we need to identify the attitude of users at a microlevel. On online social media, the individual leaning of a user i toward a specific topic, $x_{i}$, can be inferred in different ways, via the content produced or the endorsement network among users (37). Concerning content, we can define the leaning as the attitude expressed by a piece of content toward a specific topic. This leaning can be explicit (e.g., arguments supporting a narrative) or implicit (e.g., framing and agenda setting). Let us consider a user i producing a number $a_{i}$ of contents, $C_{i}=\left\{c_{1},c_{2},\ldots ,c_{a_{i}}\right\}$, where $a_{i}$ is the activity of user i, and each content leaning is assigned a numeric value. Then the individual leaning of user i can be defined as the average of the leanings of produced contents,

$$x_{i}\equiv \frac{\sum_{j=1}^{a_{i}}c_{j}}{a_{i}}.$$ [1]

Once individual leanings have been inferred, polarization can be defined as a state of the system such that the distribution of leanings, P(x), is concentrated in one or more clusters. A possible example is the case of a single cluster, distinguishable by a single, extreme peak in P(x). Another example is the typical case of topics characterized by positive versus negative stances, in which a bimodal distribution can describe polarization. For instance, if opinions are assumed to be embedded in a one-dimensional space (38), x∈[−1,+1] without loss of generality, as usual for controversial topics, then polarization is characterized by two well-separated peaks in P(x), for positive and negative opinions. In contrast, neutral ones are absent or underrepresented in the population. Note that polarization can happen independently from the structure or the very presence of social interactions. Homophily in social interactions can be quantified by representing interactions as a social network and then analyzing its structure concerning the opinions of the users (18, 39, 40). Social networks can be reconstructed in different ways from online social media, where links represent social relationships or interactions. Since we are interested in capturing the possible exchange of opinions between users, we assume links as the substrate over which information may flow. For instance, if user i follows user j on Twitter, user i can see tweets produced by user j, and there is a flow of information from node j to node i in the network. When the reconstructed network is directed, we assume the link direction points to potential influencers (opposite of information flow). Actions such as mentions or retweets may convey similar flows. In some cases, direct relations between users are not available in the data, so one needs to assume some proxy for social connections, for example, a link between two users if they comment on the same post on Facebook. Crucially, the two elements characterizing the presence of echo chambers, polarization and homophilic interactions, should be quantified independently.

### Implementation on Social Media.

This section explains how we implement the operational definitions defined above on different social media. For each medium, we detail 1) how we quantify users’ leaning, and 2) how we reconstruct how the information spread.

#### Twitter.

We consider the set of tweets posted by user i that contain links to news outlets of known political leaning. Each news outlet is associated with a political leaning score ranging from extreme left to extreme right following the *Materials and Methods* classification. We infer the individual leaning of a user, i, $x_{i}\in \left[-1,+1\right]$, by averaging the news organizations’ scores linked by user i according to Eq. **1**. We analyze three different datasets collected on Twitter related to controversial topics: gun control, Obamacare, and abortion. For each dataset, the social interaction network is reconstructed using the following relation so that there is a direct link from node i to node j if user i follows user j (i.e., the source). Henceforth, we focus on the dataset about abortion, and others are shown in *SI Appendix*.

#### Facebook.

We quantify the individual leaning of users considering endorsements in the form of likes to posts. Posts are produced by pages that are labeled in a certain number of categories, and, to each category, we assign a numerical value (e.g., Anti-Vax [+1] or Pro-Vax [–1]). Each like to a post (only one like per post is allowed) represents an endorsement for that content, which is assumed to be aligned with the leaning associated with the page. Thus, the user’s leaning is defined as the average of the content leanings of the posts liked by the user, according to Eq. **1**.

We analyze three different datasets collected on Facebook regarding a specific topic of discussion: vaccines, science versus conspiracy, and news. The interaction network is defined by considering comments. In such an interaction network, two users are connected if they cocommented on at least one post. Henceforth, we focus on the dataset about vaccines and news, and others are shown in *SI Appendix*.

#### Reddit.

The individual leaning of users is quantified similarly to Twitter by considering the links to news organizations in the content produced by the users, submissions, and comments. We build the interaction network considering comments and submissions. There exists a direct link from node i to node j if user i comments on a submission or comment by user j (we assume that i reads the comment they are replying to, which is written by j).

We analyze three datasets collected on different subreddits: the_donald, Politics, and News. In the following, we focus on the dataset collected on the Politics and the News subreddits, and others are shown in *SI Appendix*.

#### Gab.

The political leaning $x_{i}$ of user i is computed by considering the set of contents posted by user i containing a link to news outlets of a known political leaning, similarly to Twitter and Reddit. To obtain the leaning $x_{i}$ of user i, we averaged the scores of each link posted by user i according to Eq. **1**. The interaction network is reconstructed by exploiting the cocommenting relationships under posts in the same way as for Facebook. Given two users i and j, an undirected edge between i and j exists if and only if they comment under the same post.

## Comparative Analysis

In the following, we perform a comparative analysis of four different social media. We select one dataset for each social media: Abortion (Twitter), Vaccines (Facebook), Politics (Reddit), and Gab as a whole. Results for other datasets for the same medium are qualitatively similar, as shown in *SI Appendix*. We first characterize echo chambers in the networks’ topology, and then look at their effects on information diffusion. Finally, we directly compare news consumption on Facebook and Reddit.

### Polarization and Homophily in the Interaction Networks.

**Fig. 1.**
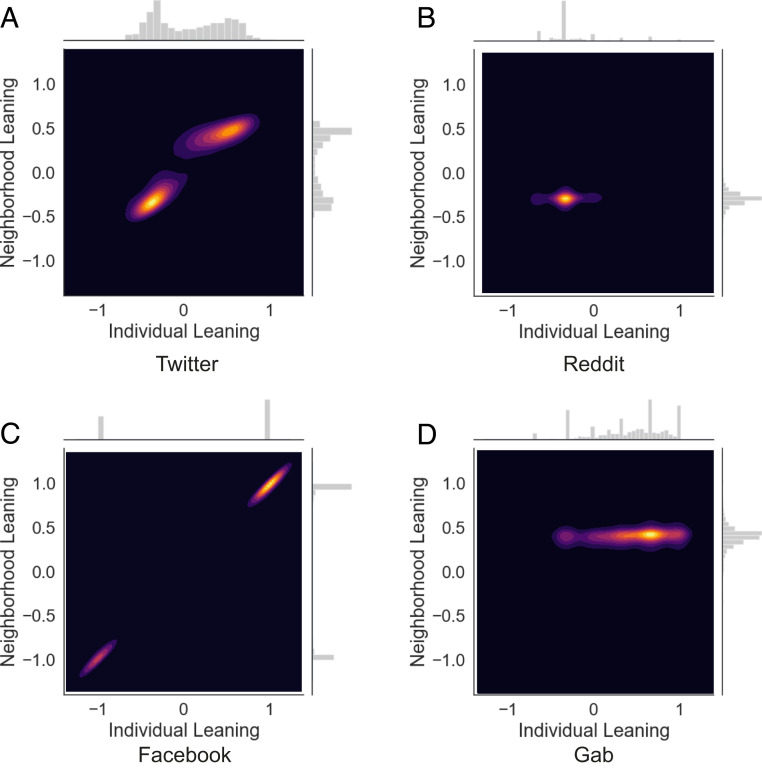
Joint distribution of the leaning of users x and the average leaning of their neighborhood $x^{NN}$ for different datasets. (*A*) Twitter, (*B*) Reddit, (*C*) Facebook, and (*D*) Gab. Colors represent the density of users: The lighter the color, the larger the number of users. Marginal distribution P(x) and $P^{N}\left(x\right)$ are plotted on the *x* and *y* axes, respectively. Facebook and Twitter present by homophilic clustering.

The network’s topology can reveal echo chambers, where users are surrounded by peers with similar leanings, and thus they get exposed, with a higher probability, to similar contents. In network terms, this translates into a node i with a given leaning $x_{i}$ more likely to be connected with nodes with a leaning close to $x_{i}$ (18). This concept can be quantified by defining, for each user i, the average leaning of their neighborhood, as $x_{i}^{N}\equiv \frac{1}{k_{i}^{\to }}\sum_{j}A_{ij}x_{j}$, where $A_{ij}$ is the adjacency matrix of the interaction network, $A_{ij}=1$ if there is a link from node i to node j, $A_{ij}=0$ otherwise, and $k_{i}^{\to }=\sum_{j}A_{ij}$ is the out-degree of node i. Fig. 1 shows the correlation between the leaning of a user i and the leaning of their neighbors, $x_{i}^{N}$, for the four social media under consideration. The probability distributions P(x) (individual leaning) and $P^{N}\left(x\right)$ (average leaning of neighbors) are plotted on the x and y axes, respectively. All plots are color-coded contour maps, representing the number of users in the phase space $\left(x,x^{N}\right)$: The brighter the area in the plan, the larger the density of users in that area. The topics of vaccines and abortion, on Facebook and Twitter, respectively, show a strong correlation between the leaning of a user and the average leaning of their nearest neighbors. Similar behavior is found for different topics from the same social media platform; see *SI Appendix*. Conversely, Reddit and Gab show a different picture. The corresponding plots in Fig. 1 display a single bright area, indicating that users do not split into groups with opposite leaning but form a single community, biased to the left (Reddit) or the right (Gab). Similar results are found for different datasets on Reddit; see *SI Appendix*.

**Fig. 2.**
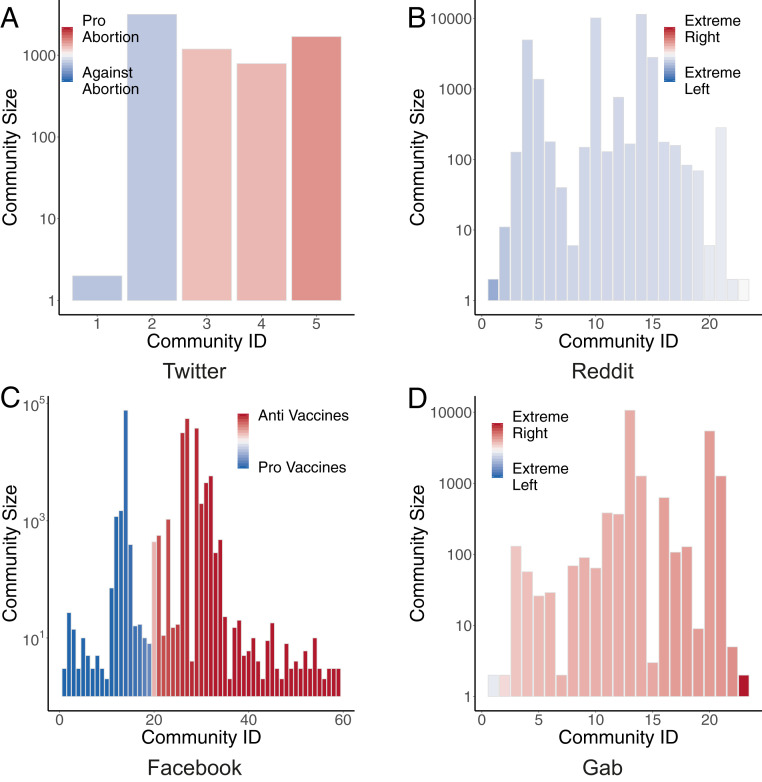
Size and average leaning of communities detected in different datasets. *A* and *C* show the full spectrum of leanings related to the topics of abortions and vaccines with regard to communities in *B* and *D*, where the political leaning is less sparse.

The presence of homophilic interactions can be confirmed by the community structure of the interaction networks. We detected communities by applying the Louvain algorithm (41), removing singleton communities with only one user. Then, we computed each community’s average leaning, determined as the average of individual leanings of its members. Fig. 2 shows the communities emerging for each social medium, arranged by increasing average leaning on the x axis (color-coded from blue to red), while the y axis reports the size of the community. On Facebook and Twitter, communities span the whole spectrum of possible leanings, but users with similar leanings form each community. Some communities are characterized by a robust average leaning, especially in the case of Facebook. These results are in accordance with the observation of homophilic interactions. Instead, communities on Reddit and Gab do not cover the whole spectrum, and all show similar average leaning. Furthermore, the almost total absence of communities with leaning very close to 0 confirms the polarized state of the systems.

### Effects on Information Spreading.

Simple models of information spreading can gauge the presence of echo chambers: Users are expected to be more likely to exchange information with peers sharing a similar leaning (18, 42, 43). Classical epidemic models such as the susceptible–infected–recovered (SIR) model (44) have been used to study the diffusion of information, such as rumors or news (45–47). In the SIR model, each agent can be in any of three states: susceptible (unaware of the circulating information), infectious (aware and willing to spread it further), or recovered (knowledgeable but not ready to transmit it anymore). Susceptible (unaware) users may become infectious (aware) upon contact with infected neighbors, with a specific transmission probability β. Infectious users can spontaneously become recovered with probability ν. To measure the effects of the leaning of users on the diffusion of information, we run the SIR dynamics on the interaction networks, by starting the epidemic process with only one node i infected, and stopping it when no more infectious nodes are left.

The set of nodes in a recovered state at the end of the dynamics started with user i as a seed of infection, that is, those that become aware of the information initially propagated by user i, form the set of influence of user i, $I_{i}$ (48). Thus, the set of influence of a user represents those individuals that can be reached by a piece of content sent by him/her, depending on the effective infection ratio β/ν. One can compute the average leaning of the set of influence of user i, $\mu_{i}$, as

$$\mu_{i}\equiv |I_{i}{|}^{-1}\underset{j\in I_{i}}{\sum }x_{j}.$$ [2]

The quantity $\mu_{i}$ indicates how polarized the users are that can be reached by a message initially propagated by user i (18).

**Fig. 3.**
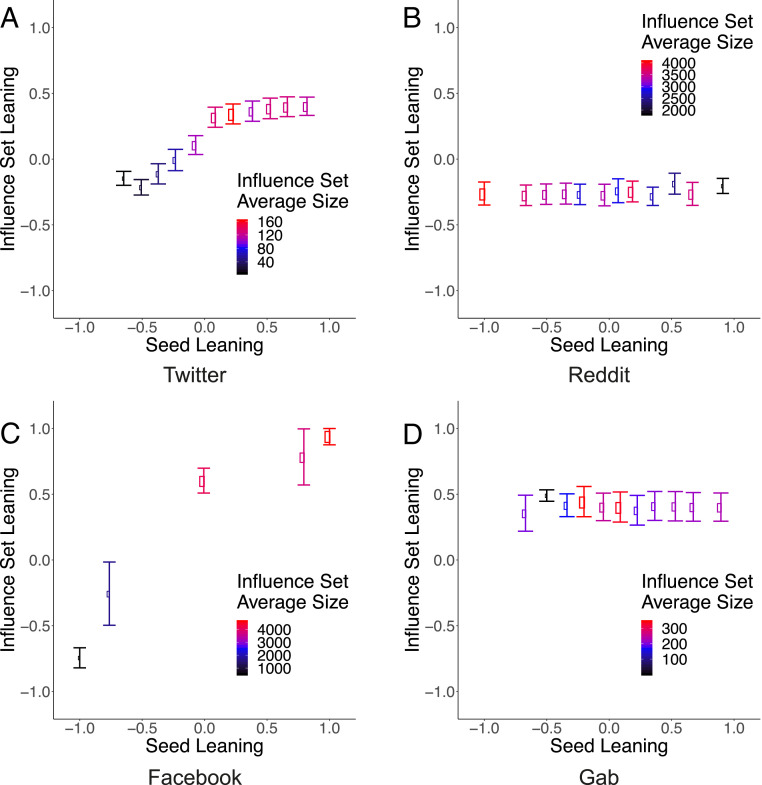
Average leaning ⟨μ(x)⟩ of the influence sets reached by users with leaning x, for different datasets under consideration. Size and color of each point represent the average size of the influence sets. The parameters of the SIR dynamics are set to (*A*) $\beta =0.10{\left\langle k\right\rangle }^{-1}$, (*B*) $\beta =0.01{\left\langle k\right\rangle }^{-1}$, (*C*) $\beta =0.05{\left\langle k\right\rangle }^{-1}$, and (*D*) $\beta =0.05{\left\langle k\right\rangle }^{-1}$, while ν is fixed at 0.2 for all simulations.

Fig. 3 shows the average leaning ⟨μ(x)⟩ of the influence sets reached by users with leaning x, for the different datasets under consideration. The recovery rate ν is fixed at 0.2 for every dataset. In contrast, the ratio between the infection rate β and average degree ⟨k⟩ depends on the specific dataset and is reported in the caption of each figure.

Again, one can observe a clear distinction between Facebook and Twitter, on one side, and Reddit and Gab on the other side. For the topics of vaccines and abortion, on Facebook and Twitter, respectively, users with a given leaning are much more likely to be reached by information propagated by users with similar leaning, that is, ⟨μ(x)⟩≈x. Similar behavior is found for different topics from the same social media platform; see *SI Appendix*. Conversely, Reddit and Gab show a different behavior: The average leaning of the set of influence, ⟨μ(x)⟩, does not depend on the leaning x. As expected, the average leaning in these media is not zero. Still, it assumes negative (positive) values in Reddit (Gab), indicating that the users of this platform are more likely to receive left (right)-leaning content.

These results indicate that information diffusion is biased toward individuals who share a similar leaning in some social media, namely Twitter and Facebook. In contrast, in others—Reddit and Gab in our analysis—this effect is absent. Such a latter configuration may depend upon two factors: 1) Gab and Reddit are not bursting the echo chamber effects, or 2) we are observing the dynamic inside a single echo chamber.

Our results are robust for different values of the effective infection ratio β/ν; see *SI Appendix*. Furthermore, Fig. 3 shows that the spreading capacity, represented by the average size of the influence sets (color-coded in Fig. 3), depends on the leaning of the users. On Twitter, proabortion users are more likely to reach larger audiences. The same is true for antivax users on Facebook, left-leaning users on Reddit, and right-leaning users on Gab (in this dataset, left-leaning users are almost absent).

### News Consumption on Facebook and Reddit.

**Fig. 4.**
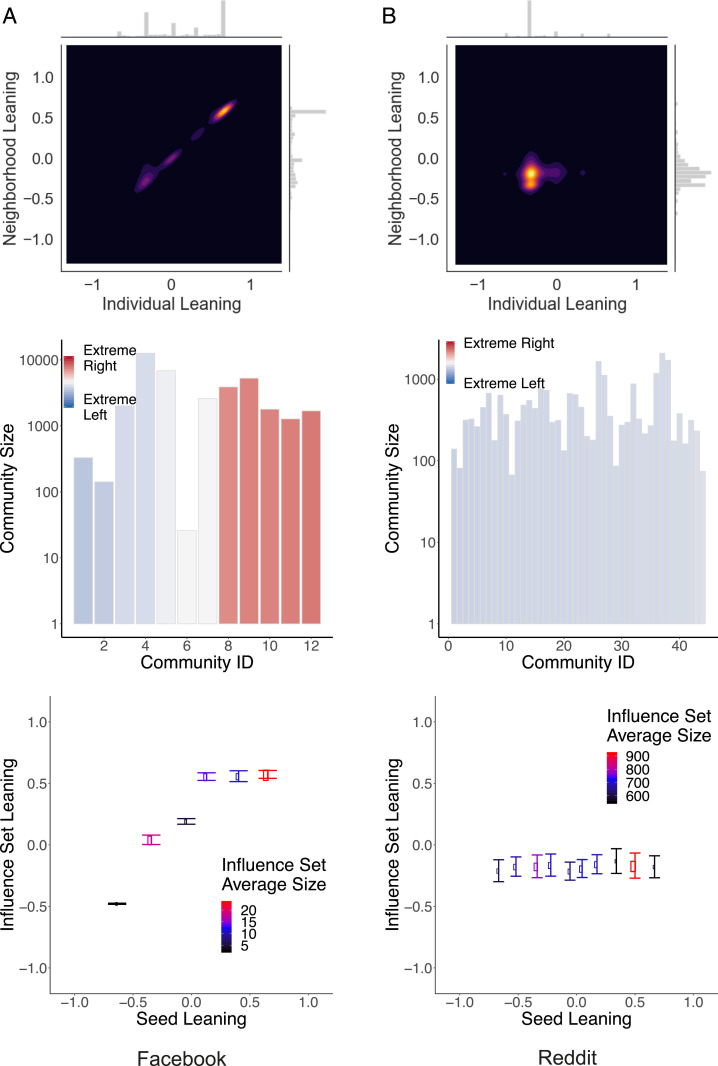
Direct comparison of news consumption on (*A*) Facebook and (*B*) Reddit. Joint distribution of the leaning of users x and the average leaning of their nearest neighbor $x^{N}$ (*Top*), size and average leaning of communities detected in the interaction networks (*Middle*), and average leaning ⟨μ(x)⟩ of the influence sets reached by users with leaning x, by running SIR dynamics (*Bottom*) with parameters β=0.05⟨k⟩ for *A*, β=0.006⟨k⟩ for *B*, and ν=0.2 for both. Facebook presents a highly segregated structure with regard to Reddit.

The striking differences observed across social media, in terms of homophily in the interaction networks and information diffusion, could be attributed to the different topics taken into account. For this reason, here we compare Facebook and Reddit on a common topic, news consumption. Facebook and Reddit are particularly apt to a cross-comparison since they share the definition of individual leaning (computed by using the classification provided by mediabiasfactcheck.org; see *Materials and Methods* for further details) and the rationale in creating connections among users that is based on an interaction network. Fig. 4 shows a direct comparison of news consumption on Facebook and Reddit along the metrics used in the previous sections to quantify the presence of echo chambers: 1) the correlation between the leaning of a user x and the average leaning of neighbors $x^{N}$ (Fig. 4, *Top*), 2) the average leaning of communities detected in the networks (Fig. 4, *Middle*), and 3) the average leaning ⟨μ(x)⟩ of the influence sets reached by users with leaning x, by running SIR dynamics (Fig. 4, *Bottom*). One can see that all three measures confirm the picture obtained for other datasets: On Facebook, we observe a clear separation among users depending on their leaning, while, on Reddit, users’ leanings are more homogeneous and show only one peak. In the latter social media, even users displaying a more extreme leaning (noticeable in the marginal histogram of Fig. 4 *B*, *Top*) tend to interact with the majority. Moreover, on Facebook, the seed user’s leaning affects who the final recipients of the information are, therefore indicating the presence of echo chambers. On Reddit, this effect is absent.

## Conclusions

Social media platforms provide direct access to an unprecedented amount of content. Platforms originally designed for user entertainment changed the way information spread. Indeed, feed algorithms mediate and influence the content promotion accounting for users’ preferences and attitudes. Such a paradigm shift affected the construction of social perceptions and the framing of narratives; it may influence policy making, political communication, and the evolution of public debate, especially on polarizing topics. Indeed, users online tend to prefer information adhering to their worldviews, ignore dissenting information, and form polarized groups around shared narratives. Furthermore, when polarization is high, misinformation quickly proliferates.

Some argued that the veracity of the information might be used as a determinant for information spreading patterns. However, selective exposure dominates content consumption on social media, and different platforms may trigger very different dynamics. In this paper, we explore the key differences between the leading social media platforms and how they are likely to influence the formation of echo chambers and information spreading. To assess the different dynamics, we perform a comparative analysis on more than 100 million pieces of content concerning controversial topics (e.g., gun control, vaccination, abortion) from Gab, Facebook, Reddit, and Twitter. The analysis focuses on two main dimensions: 1) homophily in the interaction networks and 2) bias in the information diffusion toward like-minded peers. Our results show that the aggregation in homophilic clusters of users dominates online dynamics. However, a direct comparison of news consumption on Facebook and Reddit shows higher segregation on Facebook. Furthermore, we find significant differences across platforms in terms of homophilic patterns in the network structure and biases in the information diffusion toward like-minded users. A clear-cut distinction emerges between social media having a feed algorithm tweakable by the users (e.g., Reddit) and social media that don’t provide such an option (e.g., Facebook and Twitter). Our work provides important insights into the understanding of social dynamics and information consumption on social media. The next envisioned step addresses the temporal dimension of echo chambers, to understand better how different social feedback mechanisms, specific to distinct platforms, can impact their formation.

## Materials and Methods

Here we provide details about the labeling of news outlets and the datasets considered.

### Labeling of Media Sources.

The labeling of news outlets is based on the information reported by Media Bias/Fact Check (MBFC) (https://mediabiasfactcheck.com), an independent fact-checking organization that rates news outlets on the basis of the reliability and of the political bias of the contents they produce and share. The labeling provided by MBFC, retrieved in June 2019, ranges from Extreme Left to Extreme Right for political bias. The total number of media outlets for which we have a political label is 2,190. A detailed description of the source labeling process and political bias distribution can be found in *SI Appendix*.

### Data Availability.

For what concerns Gab, all data are available on the Pushshift public repository (https://pushshift.io/what-is-pushshift-io/) at this link: https://files.pushshift.io/gab/. Reddit data are available on the Pushshift public repository at this link: https://search.pushshift.io/reddit/. For what concerns Facebook and Twitter, we provide data according to their Terms of Services on the corresponding author institutional page at this link: https://walterquattrociocchi.site.uniroma1.it/ricerca. For news outlet classification, we used data from MBFC (https://mediabiasfactcheck.com), an independent fact-checking organization. Anonymized data have been deposited in Open Science Framework (10.17605/OSF.IO/X92BR) (49). For further details about data, refer to the following section.

### Empirical Datasets.

Table 1 reports summary statistics of the datasets under consideration. Due to the structural differences among platforms, each dataset has different features. For Twitter, we used tweets regarding three topics collected by Garimella et al. (16), namely Gun control, Obamacare, and abortion. Tweets linking to a news source with a known bias are classified based on MBFC. Facebook datasets were created by using Facebook Graph API and were previously explored in ref. 50 (Science and Conspiracy), (51) (Vaccines) and (11) (News). For the two datasets Science and Conspiracy and Vaccines, data were labeled in a binary way, respectively, provaccines/antivaccines and proscience/conspiracy, based on the page where they were posted. Posts in the dataset News were instead classified based on MBFC labeling. Reddit datasets have been obtained by downloading comments and submissions posted in the subreddit Politics, the_donald, and News and labeled according to the classification obtained from MBFC. The Gab dataset has been collected from https://files.pushshift.io/gab and contains posts, replies, and quotations. Posts were labeled according to MBFC classification. Further details can be found in *SI Appendix*.

**Table 1.** Dataset details

Media	Dataset	$T_{0}$	T	C	N	$n_{c}$
Twitter	Gun control	June 2016	14 d	19 million	3,963	0.93
	Obamacare	June 2016	7 d	39 million	8,703	0.90
	Abortion	June 2016	7 d	34 million	7,401	0.95
Facebook	Sci/Cons	January 2010	5 y	75,172	183,378	1.00
	Vaccines	January 2010	7 y	94,776	221,758	1.00
	News	January 2010	6 y	15,540	38,663	1.00
Reddit	Politics	January 2017	1 y	353,864	240,455	0.15
	the_donald	January 2017	1 y	1.234 million	138,617	0.16
	News	January 2017	1 y	723,235	179,549	0.20
Gab	Gab	November 2017	1 y	13 million	165,162	0.13

For each dataset, we report the starting date of collection $T_{0}$, time span T expressed in days (d) or years (y), number of unique contents C, number of users N, and coverage $n_{c}$ (fraction of users with classified leaning). For Twitter, T represents the window to sample active users, of which we retrieve all of the tweets related to the topic via the Application Programming Interface (API) (more information in *SI Appendix*). Sci/Cons, Scientific and Conspiracy content.

## Supplementary Material

Supplementary File
