# Supplementary material for: The echo chamber effect on social media
Source: Proc Natl Acad Sci U S A. 2021 Feb 23;118(9):e2023301118. doi: 10.1073/pnas.2023301118 (PMC7936330; doi:10.1073/pnas.2023301118)
Supplement: Supplementary File [file pnas.2023301118.sapp.pdf]

Supplementary Information for

The echo chamber effect on social media

Matteo Cinelli,Gianmarco De Francisci Morales, Alessandro Galeazzi, Walter Quattrociocchi, Michele Starnini

Corresponding Walter Quattrociocchi.
E-mail: walter.quattrociocchi@uniroma1.it

This PDF file includes:

Supplementary text
Figs. S1 to S7
Table S1
SI References

Supporting Information Text

Here we provide more details concerning the labelling of news outlets, employed datasets and we provide additional results that were omitted in the main paper for the sake of brevity. The description of the labelling process is reported in Section 1, the detailed datasets description is reported in Section 2, further results on datasets that were omitted from the main text are reported in Section 3 while results for different parametrization of the SIR model are reported in Section 4.

1. Labelling of Media Sources

The labelling of news outlets is based on the information provided by Media Bias/Fact Check (MBFC https://mediabiasfactcheck.com), an independent fact-checking organization that rates news outlets on the base of the reliability and of the political bias of the contents they produce and share. The website provides the political bias related to a wide range of media outlets. The labelling provided by MBFC, retrieved in June 2019, ranges from Extreme Left to Extreme Right for what concerns the political bias. Moreover, certain media outlets are classified as 'questionable' sources or 'conspiracy-pseudoscience' sources if they tend to publish misinformation or false contents. Often, such news outlets (without an explicit political label reported by MBFC) actually display a political bias that is reported in their description, as shown in Figure S1.

Considering the importance of including such media outlets in our analysis, we manually reported their classification from the description provided by MBFC, thus adding 468 outlets to the pool of 1722 news outlets that already have a clear political label. The total number of labelled news outlets is 2190 and the overall leaning is summarized in Figure S2. In order to compute the individual leaning of users we convert each label into a numerical value, namely, -1 for Extreme Left, -0.66 for Left, -0.33 for Left-Center, 0 for Least Biased, 0.33 for Right-Center, 0.66 for Right and +1 for Extreme Right.

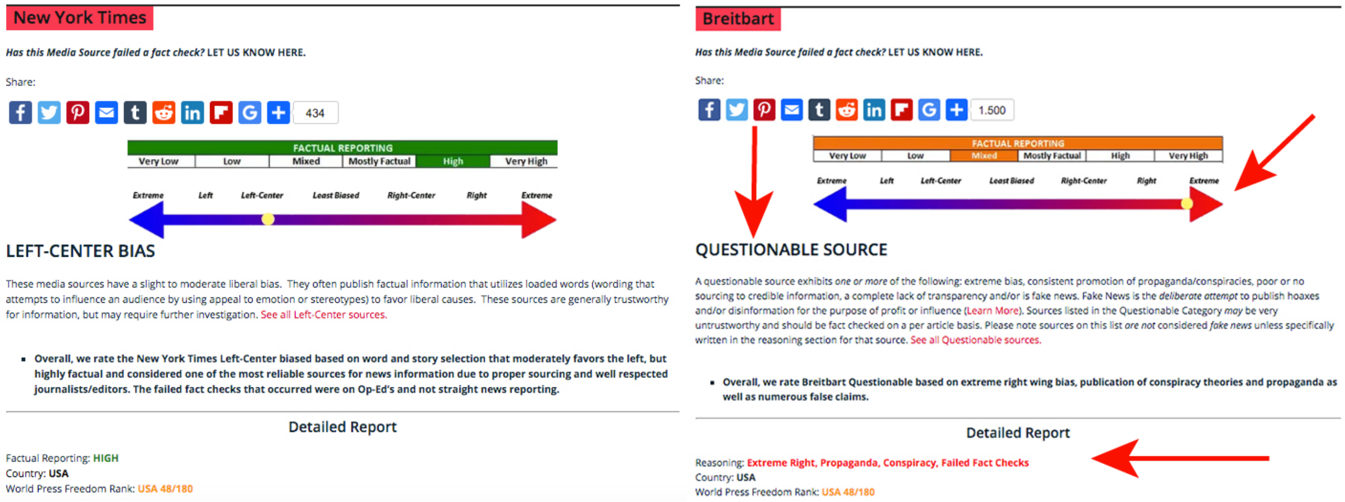

Fig. S1. Example of the web page of MBFC for two news outlets, namely New York Time and Breitbart. Notice that, although Breitbart is labeled as "Questionable", an explicit leaning appears in its description.

2. Dataset detailed description

Here we report details on data collection for different social media, as summarized in Table S1.

Twitter. We follow a two-step procedure for creating the Twitter datasets. First, tweets during the interest periods are retrieved from the Internet Archive Twitter Stream.* For each topic, we use the keywords specified by Lu et al. (1). Each user that has

*https://archive.org/details/twitterstream

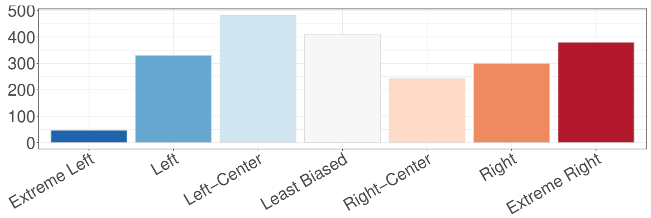

Fig. S2. Distribution of the leanings assigned to each source, ranging from Extreme Left (numerical value: -1, colored in blue) to Extreme Right (numerical value: +1, colored in red).

Table S1. For each data set, we report: the starting date of collection T_0, time span T expressed in days (d) or years (y), number of unique contents C, number of users N, coverage n_c (fraction of users with classified leaning), size of the giant component G and average node degree $\langle k \rangle$.

Media	Data set	T_0	T	C	N	n_c	G	$\langle k \rangle$
	Gun control	06/2016	14 d	19 M	3963	0.93	3717	798
Twitter	Obamacare	06/2016	7 d	39 M	8703	0.90	8703	1405
	Abortion	06/2016	7 d	34 M	7401	0.95	6828	478
	Sci/Cons	01/2010	5 y	75 172	183 378	1.00	181960	228
Facebook	Vaccines	01/2010	7 y	94 776	221 758	1.00	220275	419
	News	01/2010	6 y	15 540	38 663	1.00	38594	700
	Politics	01/2017	1 y	353 864	240 455	0.15	240455	9
Reddit	The Donald	01/2017	1 y	1.234 M	138 617	0.16	138617	31
	News	01/2017	1 y	723 235	179 549	0.20	179549	3
Gab	Gab	11/2017	1 y	13 M	165 162	0.13	20701	328

posted 5 or more tweets on the topic during the window of interest is considered active. We then use the Twitter's REST API[†] to collect all tweets and followers for each active user. These tweets and relationships are the basis for reconstructing each network. For more info on the datasets, see the work by Garimella et al. (2).

Gun control. The interest window spans 14 days in June 2016. We consider $C = 19$M tweets produced by $N = 7506$ users. We reconstruct a directed follow network formed by $E = 1\,053\,275$ directed edges. The largest weakly connected component includes more than 99% of nodes. We identify the individual leaning of $N_c = 6994$ users.

Obamacare. The interest window spans 7 days in June 2016. We consider $C = 34$M tweets produced by $N = 8773$ users. We reconstruct a directed follow network formed by $E = 3\,797\,871$ directed edges. The largest weakly connected component includes more than 99% of nodes. We identify the individual leaning of $N_c = 7899$ users.

Abortion. The interest window spans 7 days in June 2016. We consider $C = 34$M tweets produced by $N = 3995$ users. We reconstruct a directed follow network formed by $E = 2\,330\,276$ directed edges. The largest weakly connected component includes more than 99% of nodes. We identify the individual leaning of $N_c = 3809$ users.

Facebook.

Science and Conspiracy. The dataset was built by downloading posts of selected Facebook pages divided into two groups, namely conspiracy news and science news. Conspiracy pages were selected based on their name, their self description and with the aid of debunking pages. The selection process was iterated until convergence among annotators. The dataset, that includes post from pages and comments to such posts, was created by using Facebook Graph API and has been previously explored (3). We consider 75 172 posts by 73 pages categorized in Science (34) and Conspiracy (39) that involve $N = 183\,378$ active users (at least 1 like and 1 comments), for which we identify the individual leaning, that co-commented 20 807 976 times. Using this dataset we build an undirected network, where two users (nodes) are connected if and only if they commented under the same post at least once. The largest connected component of the co-commenting network has $G = 181\,960$ nodes and $E = 20\,807\,491$ links.

Vaccines. The dataset was generated in three steps: first a search for pages containing the keywords vaccine, vaccines, or vaccination was made. Then the raw outcome was cleaned from spurious pages. Finally, all the posts and comments of selected pages were downloaded and pages were manually classified in Pro-Vax and Anti-Vax groups. The dataset was created by using Facebook Graph API and has been previously explored (4). We consider 94 776 posts by 243 pages categorized in Pro-Vax (145) and Anti-Vax (98) that involve 221 758 active users (at least 1 like and 1 comment), for which we identify the individual leaning, that co-commented 46 198 446 times. Using this dataset we build an undirected network, where two users (nodes) are connected if and only if they commented under the same post at least once. The largest connected component of the co-commenting network has $G = 220\,275$ nodes and $E = 46\,193\,632$ links.

News. The dataset was built by considering a set of Facebook pages of news outlets listed by the Europe Media Monitor. By using the Facebook Graph API, all the posts and comments related to these pages in the period 2010-2015 were downloaded. Facebook pages are labelled according to the annotation obtained by MBFC. The dataset without annotations has been previously explored (5). We consider 15 540 posts by 180 pages categorized from Left to Right (Left (12), Left-Center (80), Least-Biased (42), Right-Center (33), Right (13)). Such posts were co-commented 13 525 230 times by 38663 active users (users with at least 3 likes and 3 comments), for which we identify the individual leaning. Using this dataset we build a undirected network, where two users (nodes) are connected if and only if they commented under the same post at least once. The largest connected component of the co-interaction network has $G = 38\,594$ nodes and $E = 13\,525\,119$ links.

Reddit.

[†] https://developer.twitter.com/en/docs/twitter-api/v1

Politics. We consider 353 864 comments and submissions posted on the subreddit *politics* in the year 2017. From comments under submissions we reconstructed a directed network formed by $N = 240\,455$ users and $E = 5\,030\,565$ directed edges, where each edge represents a direct reply to a comment. The largest weakly connected component includes more than 99% of nodes. We exploited the classification retrieved from MBFC to identify the individual leaning of $N_c = 37\,148$ users, that is considered as a scalar feature of the node.

The Donald. We consider 1.234M comments and submissions posted on the subreddit *The_Donald* in the year 2017. From comments a submissions we reconstructed a directed network formed by $N = 138\,617$ users and $E = 5\,025\,290$ directed edges, where each edge represents a direct reply to a comment. The largest weakly connected component includes more than 99% of nodes. We exploited the classification retrieved from MBFC to identify the individual leaning of $N_c = 21\,905$ users.

News. We consider 723 235 comments and submissions posted on the subreddit *news* in the year 2017. From comments a submissions we reconstructed a directed network formed by $N = 179\,549$ users and $E = 1\,070\,589$ directed edges, where each edge represents a direct reply to a comment. The largest weakly connected component includes more than 99% of nodes. We exploited the classification retrieved from MBFC to identify the individual leaning of $N_c = 36\,875$ users.

Gab. The dataset, downloaded from https://files.pushshift.io/gab, spans from the first Gab post (occurred in 2016) to the late 2018 and it includes data regarding post-reply relationships, number of upvotes of posts, repost or replies and their timestamps. We selected all the contents (post, reply, quote) in the time window ranging from 11/2017 to 10/2018, that is $C = 13\,580\,937$ unique pieces of content created by $N = 165\,162$ unique users. We consider all the post that have a link to an external source, for an amount of 3 302 621 posts (excluding YouTube links). By extracting the domain from each link we obtain a set of 75 436 unique domains. In this set, 1650 unique domains for a total of 1 454 502 URLs (44%) were labelled using the classification provided by MBFC. We identified the individual leaning of $N_c = 31\,286$ users.We also reconstructed the interaction network using co-commenting as a proxy, that is, two users are connected if and only if they commented under the same post at least once. The largest connected component of the network includes $G = 20\,701$ nodes, about 66% of the users with assigned leaning, and $E = 8\,273\,412$ edges. The individual leaning x_i is considered as a scalar feature of the node.

3. Analysis for other datasets

In this section we report the results obtained for other four data sets not shown in the main paper, namely "Science and Conspiracy" (Facebook), "Gun control" (Twitter), "Obamacare" (Twitter) and 'The Donald" (Reddit). The techniques and the pipeline is the same used for the datasets analyzed in the main paper.

A. Science and Conspiracy. Figure S3 displays the results obtained for the Facebook dataset called "Science and Conspiracy", described in Section 2. Panel (a) shows the joint distribution of the leaning of users, x, against the average leaning of their neighborhood X^N. We note that the community referred to as "Science", to which is associated a leaning of -1, is much smaller than the community called "Conspiracy" and for this reason it is not clearly visible in the density plot but only in the histograms at its margins. Panel (b) shows the size and average leaning of communities detected by the Louvain algorithm.

Panels (c) and (d) show the results of the SIR dynamics: the average leaning $\langle \mu(x) \rangle$ of the influence sets reached by users with leaning x, for two different values of the infection probability, while the recovery rate is fixed $\nu = 0.2$. Size and color of each point is related to the average size of the influence sets.

B. Guncontrol. Figure S4 shows the results obtained for the Twitter dataset "Gun control", described in Section 2. Panel (a) shows the joint distribution of the leaning of users, x, against the average leaning of their neighborhood X^N, in which two different regions are clearly visible. Panel (b) shows the size and average leaning of communities detected by the Louvain algorithm.

Panels (c) and (d) show the results of the SIR dynamics: the average leaning $\langle \mu(x) \rangle$ of the influence sets reached by users with leaning x, for two different values of the infection probability, while the recovery rate is fixed $\nu = 0.2$. Size and color of each point is related to the average size of the influence sets.

C. Obamacare. Figure S5 shows the results obtained for the Twitter dataset referred to as "Obamacare", described in Section 2. Panel (a) shows the joint distribution of the leaning of users, x, against the average leaning of their neighborhood X^N, in which two interconnected regions are clearly visible. Panel (b) shows the size and average leaning of communities detected by the Louvain algorithm.

Panels (c) and (d) show the results of the SIR dynamics: the average leaning $\langle \mu(x) \rangle$ of the influence sets reached by users with leaning x, for two different values of the infection probability, while the recovery rate is fixed $\nu = 0.2$. Size and color of each point is related to the average size of the influence sets.

D. TheDonald. Figure S6 shows the results obtained for the Reddit dataset "The Donald", described in Section 2. Panel (a) displays the joint distribution of the leaning of users, x, against the average leaning of their neighborhood X^N, showing a unique region spanning most of the x-axis and concentrated on the values around 0.25 on the y-axis. Such a region is also characterized by few peaks of leaning (spanning mainly from Center to Extreme Right) that are displayed in the histogram on the top margin. Panel (b) shows the size and average leaning of communities detected by the Louvain algorithm.

Panels (c) and (d) show the results of the SIR dynamics: the average leaning $\langle \mu(x) \rangle$ of the influence sets reached by users with leaning x, for two different values of the infection probability, while the recovery rate is fixed $\nu = 0.2$. Size and color of each point is related to the average size of the influence sets.

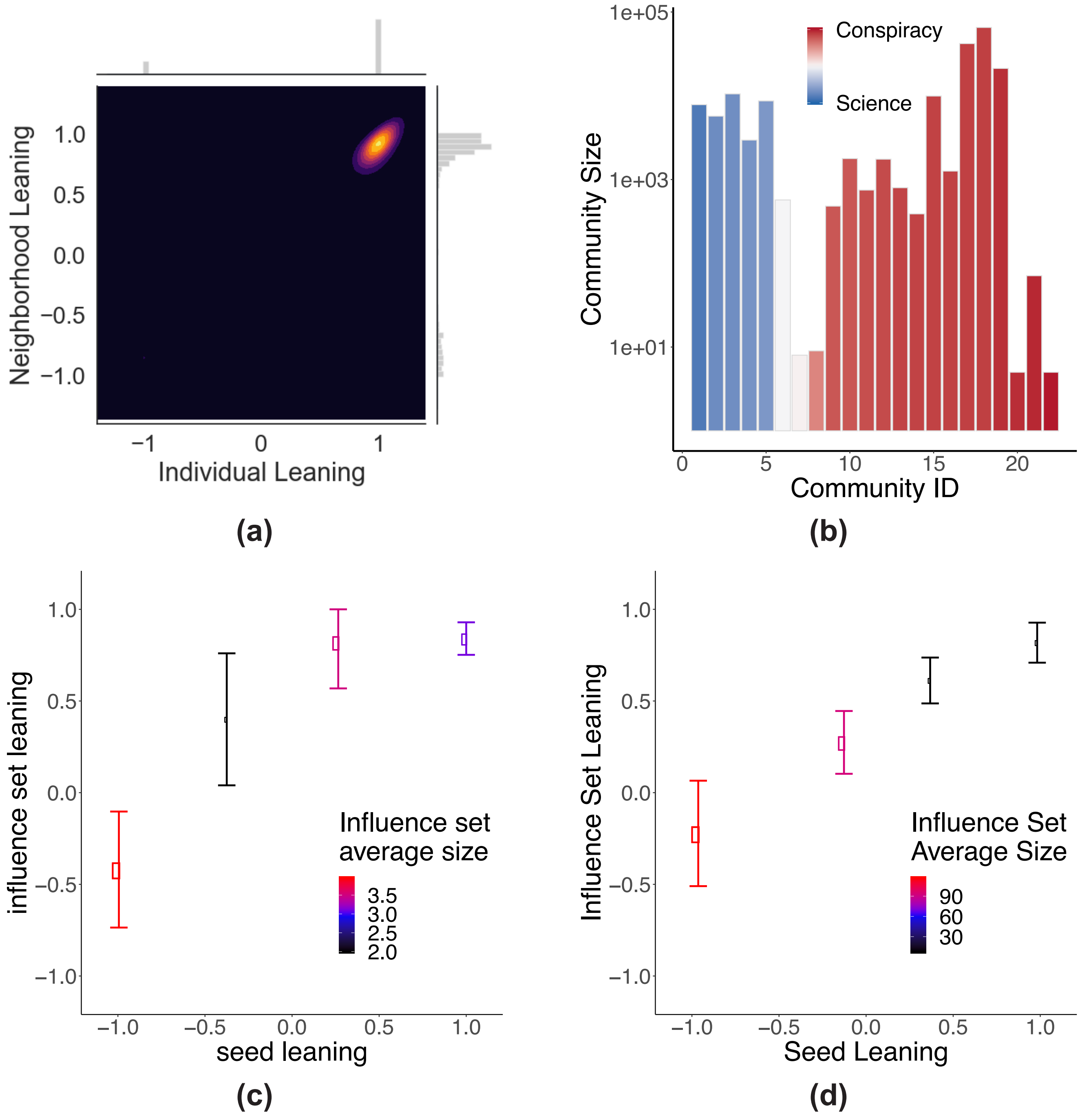

Fig. S3. Science vs Conspiracy. Panel (a): Individual leaning versus neighborhood leaning. Panel (b): Community detection. Panel (c) and (d): average leaning $\langle \mu(x) \rangle$ of the influence sets reached by users with leaning x, for infection probability $\beta = 0.01\langle k \rangle^{-1}$ and $\beta = 0.02\langle k \rangle^{-1}$, respectively, where $\langle k \rangle$ is the average degree of the network.

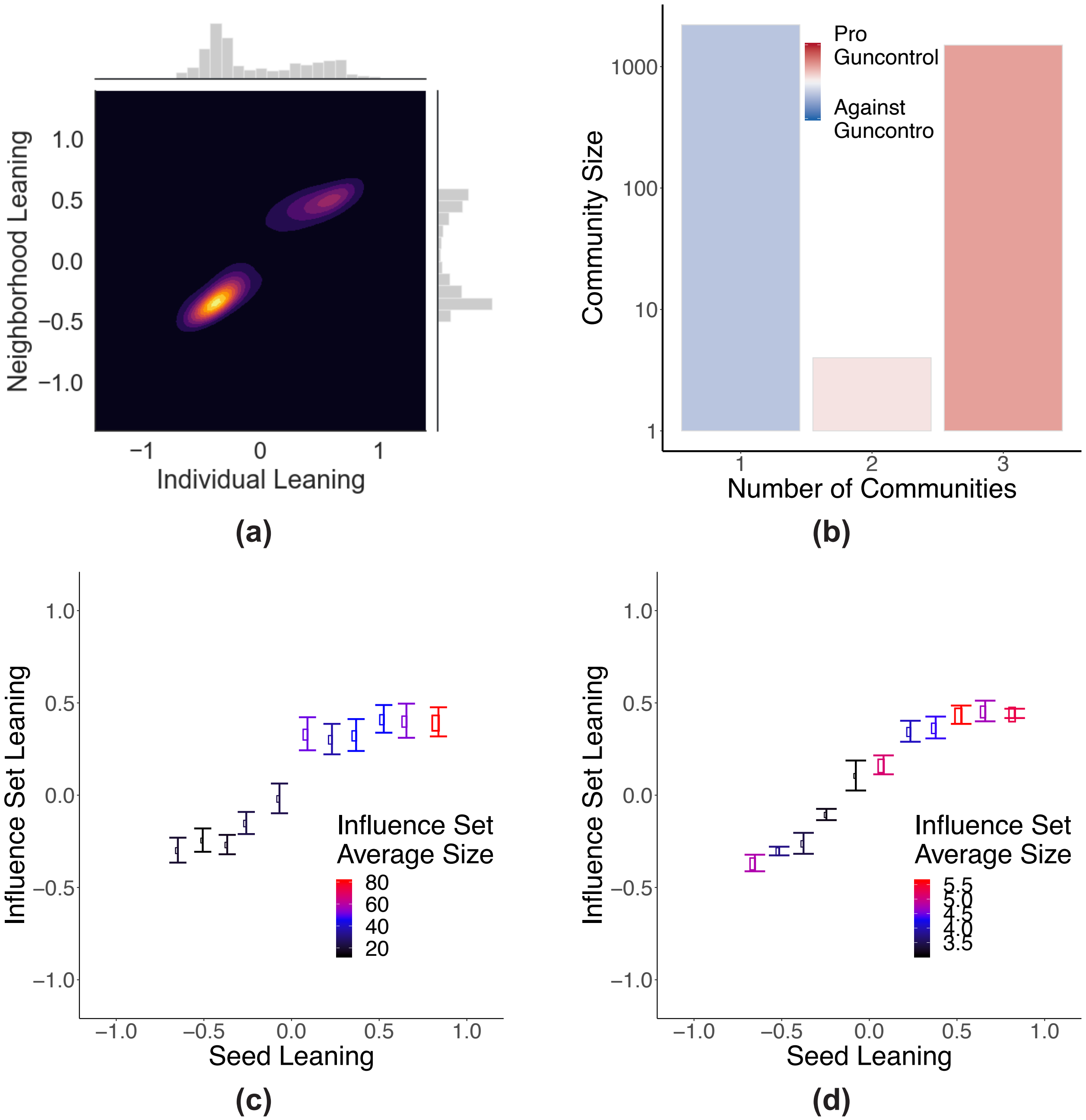

Fig. S4. Gun control. Panel (a): Individual leaning versus neighborhood leaning. Panel (b): Community detection. Panel (c) and (d): average leaning $\langle \mu(x) \rangle$ of the influence sets reached by users with leaning x, for infection probability $\beta = 0.1\langle k \rangle^{-1}$ and $\beta = 0.2\langle k \rangle^{-1}$, respectively, where $\langle k \rangle$ is the average degree of the network.

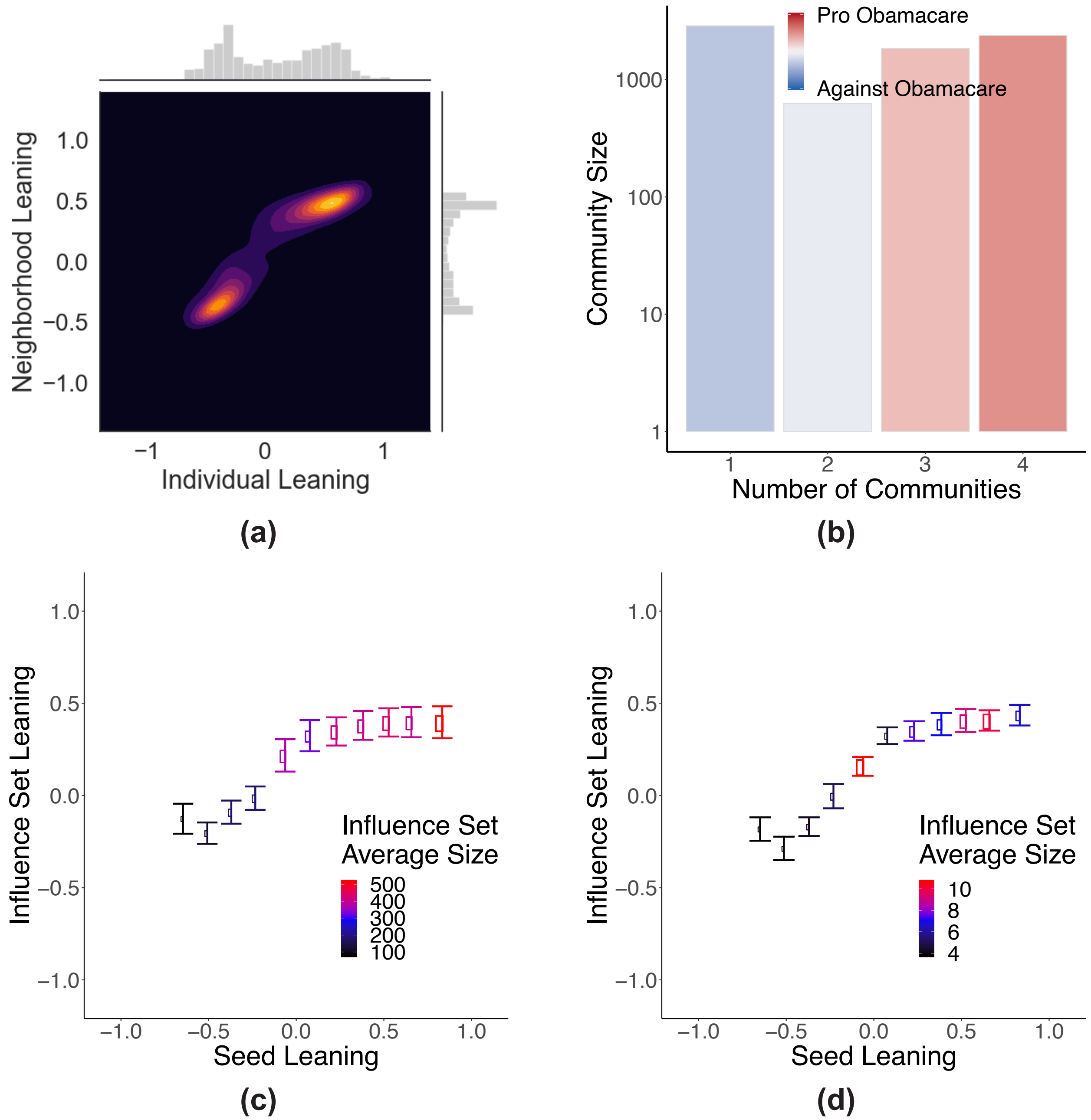

Fig. S5. Obamacare. Panel (a): Individual leaning versus neighborhood leaning. Panel (b): Community detection. Panel (c) and (d): average leaning $\langle \mu(x) \rangle$ of the influence sets reached by users with leaning x, for infection probability $\beta = 0.1\langle k \rangle^{-1}$ and $\beta = 0.2\langle k \rangle^{-1}$, respectively, where $\langle k \rangle$ is the average degree of the network.

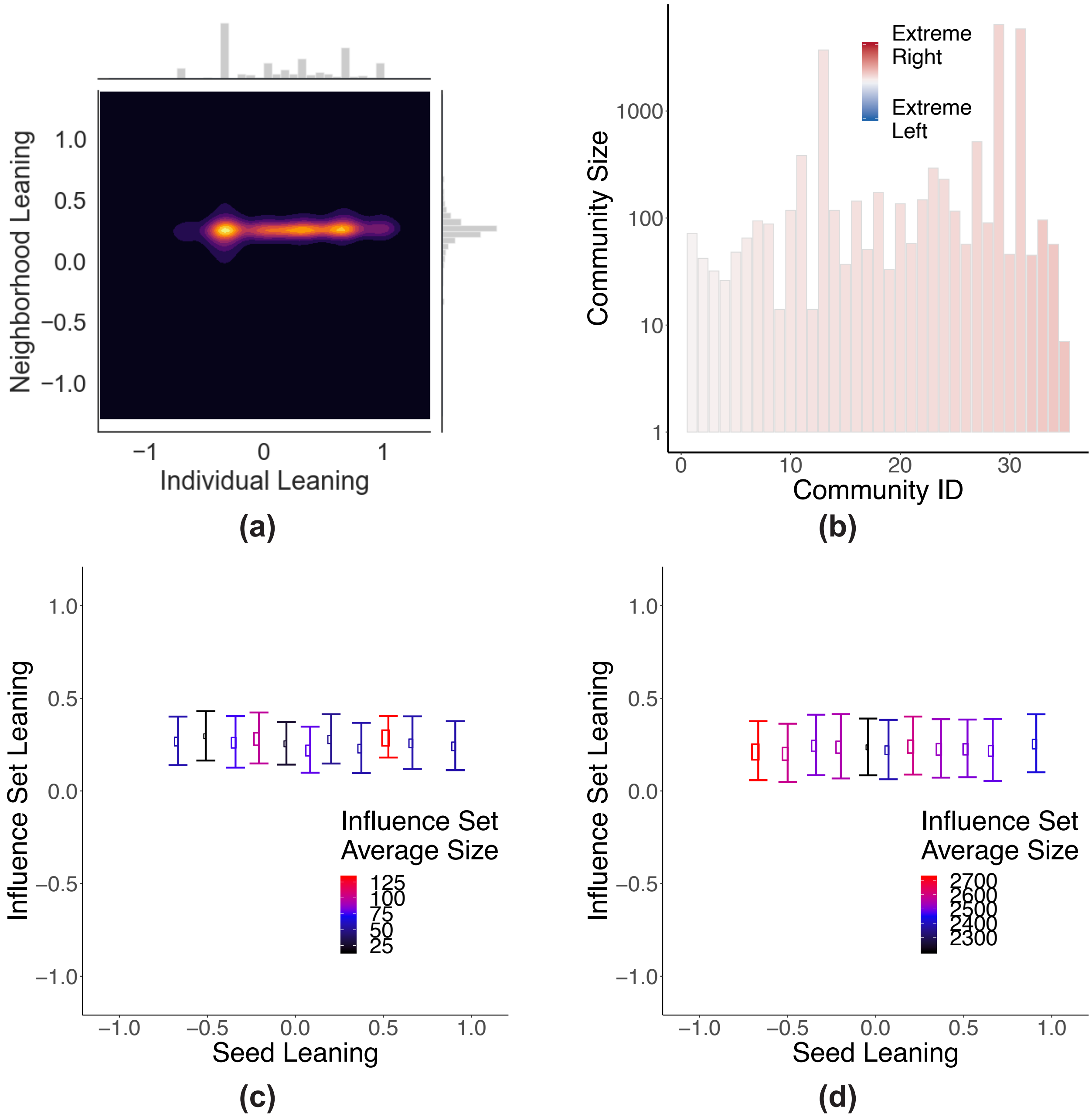

Fig. S6. The Donald. Panel (a): Individual leaning versus neighborhood leaning. Panel (b): Community detection. Panel (c) and (d): average leaning $\langle\mu(x)\rangle$ of the influence sets reached by users with leaning x, for infection probability $\beta = 0.0067\langle k\rangle^{-1}$ and $\beta = 0.013\langle k\rangle^{-1}$, respectively, where $\langle k\rangle$ is the average degree of the network.

4. Robustness of the SIR dynamics

In this section, we provide additional results for the SIR dynamics run with different parameters on the 6 data sets considered in the main paper, namely "Abortion" on Twitter, "Politics" and "News" on Reddit, "Vaccines" and "News" on Facebook, and Gab.

The results, reported in Fig. S7, are qualitatively identical to the ones in the main paper and are reported here for the sake of brevity. Details about the parameters used in the simulations are provided in the caption of Fig. S7.

References

1. H Lu, J Caverlee, W Niu, Biaswatch: A lightweight system for discovering and tracking topic-sensitive opinion bias in social media in *CIKM.* (ACM), pp. 213–222 (2015).
2. K Garimella, G De Francisci Morales, A Gionis, M Mathioudakis, Political discourse on social media: Echo chambers, gatekeepers, and the price of bipartisanship in *Proceedings of the 2018 World Wide Web Conference*, WWW '18. (International World Wide Web Conferences Steering Committee, Republic and Canton of Geneva, Switzerland), pp. 913–922 (2018).
3. A Bessi, et al., Users polarization on facebook and youtube. *PloS one* **11**, e0159641 (2016).
4. AL Schmidt, F Zollo, A Scala, C Betsch, W Quattrociocchi, Polarization of the vaccination debate on facebook. *Vaccine* **36**, 3606–3612 (2018).
5. AL Schmidt, et al., Anatomy of news consumption on facebook. *Proc. Natl. Acad. Sci.* **114**, 3035–3039 (2017).

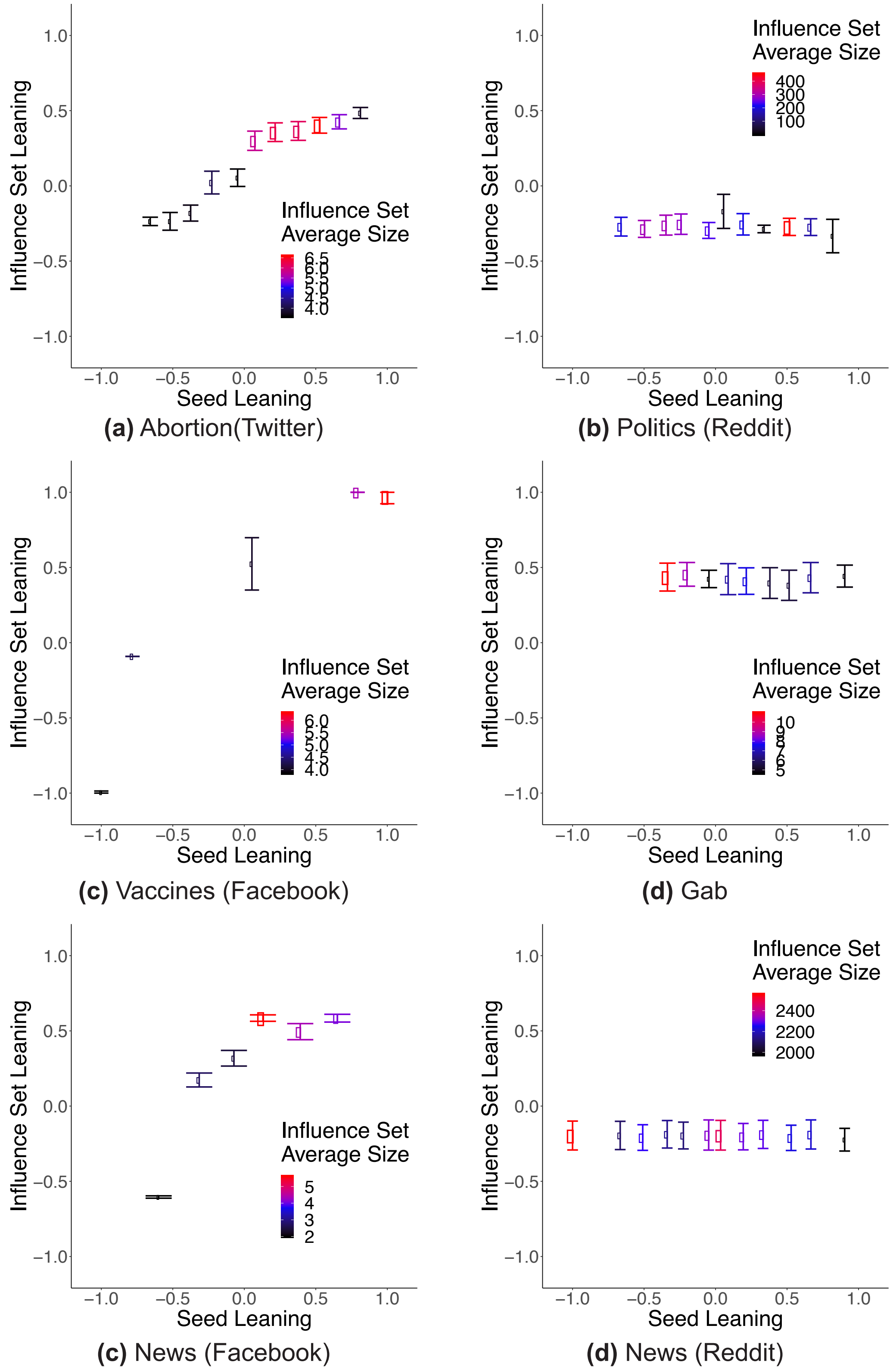

Fig. S7. Additional results of the SIR dynamics for the six data sets considered in the main paper. Average leaning $\langle\mu(x)\rangle$ of the influence sets reached by users with leaning x, for infection probability $\beta = 0.05\langle k\rangle^{-1}$ (Abortion on Twitter, panel (a)), $\beta = 0.005\langle k\rangle^{-1}$ (Politics on Reddit, panel (b)), $\beta = 0.02\langle k\rangle^{-1}$ (Vaccines on Facebook, panel (c)), $\beta = 0.025\langle k\rangle^{-1}$ (Gab, panel (d)), $\beta = 0.025\langle k\rangle^{-1}$ (News on Facebook, panel (e)), $\beta = 0.01\langle k\rangle^{-1}$ (News on Reddit, panel (f)), while the recovery rate is fixed $\nu = 0.2$. Size and color of each point is related to the average size of the influence sets.
